# Digital Platform for Automatic Qualitative and Quantitative Reading of a Cryptococcal Antigen Point-of-Care Assay Leveraging Smartphones and Artificial Intelligence

**DOI:** 10.3390/jof9020217

**Published:** 2023-02-07

**Authors:** David Bermejo-Peláez, Narda Medina, Elisa Álamo, Juan Carlos Soto-Debran, Oscar Bonilla, Miguel Luengo-Oroz, Juan Luis Rodriguez-Tudela, Ana Alastruey-Izquierdo

**Affiliations:** 1 Spotlab, 28040 Madrid, Spain; 2 Mycology Reference Laboratory, National Center for Microbiology, Instituto de Salud Carlos III, 28220 Madrid, Spain; 3 Asociación de Salud Integral, Guatemala City 01001, Guatemala; 4 Clínica Familiar “Luis Ángel García”, Hospital General San Juan de Dios, Guatemala City 01001, Guatemala; 5 Global Action for Fungal Infections, 1208 Geneva, Switzerland; 6 Centro de Investigación Biomédica en Red de Enfermedades Infecciosas, Instituto de Salud Carlos III, 28029 Madrid, Spain

**Keywords:** lateral flow assay (LFA), rapid diagnostic test (POCT), smartphone, artificial intelligence (AI), *Cryptococcus*, cryptococcal antigen, test line quantification

## Abstract

Cryptococcosis is a fungal infection that causes serious illness, particularly in immunocompromised individuals such as people living with HIV. Point of care tests (POCT) can help identify and diagnose patients with several advantages including rapid results and ease of use. The cryptococcal antigen (CrAg) lateral flow assay (LFA) has demonstrated excellent performance in diagnosing cryptococcosis, and it is particularly useful in resource-limited settings where laboratory-based tests may not be readily available. The use of artificial intelligence (AI) for the interpretation of rapid diagnostic tests can improve the accuracy and speed of test results, as well as reduce the cost and workload of healthcare professionals, reducing subjectivity associated with its interpretation. In this work, we analyze a smartphone-based digital system assisted by AI to automatically interpret CrAg LFA as well as to estimate the antigen concentration in the strip. The system showed excellent performance for predicting LFA qualitative interpretation with an area under the receiver operating characteristic curve of 0.997. On the other hand, its potential to predict antigen concentration based solely on a photograph of the LFA has also been demonstrated, finding a strong correlation between band intensity and antigen concentration, with a Pearson correlation coefficient of 0.953. The system, which is connected to a cloud web platform, allows for case identification, quality control, and real-time monitoring.

## 1. Introduction

Cryptococcosis is a life-threatening invasive fungal infection, with mortality ranging between 41 and 61%, especially in people living with HIV (PLWHIV) with meningitis being the most serious of its multiple manifestations. However, it has also been described in other immunocompromised hosts and as well as in people without known risk factors. An estimated 152,000 cases of cryptococcal meningitis occur each year, most of them in sub-Saharan Africa [1]. WHO guidelines recommend serum cryptococcal antigen (CrAg) screening for all patients with advanced HIV disease followed by a lumbar puncture when feasible for those testing positive to diagnose or rule out cryptococcal meningitis. High titers of antigens are associated with meningitis and death [2,3,4].

The cryptococcal antigen lateral flow assay (CrAg LFA, IMMY, OK, USA) is a point-of-care test (POCT) with sensitivity and specificity >99% and 98% respectively [5]. Moreover, this assay has several technical advantages as it has a rapid turnaround time, requires little training for use, and can be performed with minimal laboratory infrastructure, which has resulted in an important tool, especially for public health programs that look to reduce HIV-related mortality. However, a limitation of the use of LFAs is that results can be subject to individual interpretation, potentially leading to inconsistent or biased conclusions.

The widespread availability and use of smartphones have powerful implications for digital and mobile health care. The use of these devices in conjunction with POCTs would offer tremendous potential for improving the access to diagnosis, particularly in low-resource countries [6,7]. Mobile phones are the backbone of development for many tasks that previously were carried out through computers. Their connection with the Internet and cloud services make them a key tool to develop and use artificial intelligence (AI)-based apps able to automatically interpret the results of POCTs in an objective manner, as well as to immediately transfer the results to the clinicians in charge of the patient to establish effective treatment. In addition, the results can be kept in observational big databases that can ascertain the local epidemiology of the diagnosed disease as well as perform other tasks able to understand the real management of the fungal disease in the studied environment. Smartphone-based systems have already demonstrated their capacity to automatically interpret POCT results for different diseases including HIV, SARS-CoV-2, and malaria among others, performing both qualitative [8,9,10,11,12,13] and quantitative [14,15,16,17,18] analyses. However, most of these approaches rely on the use of additional hardware to be robust among different ambient lighting conditions or to acquire standardized POCT pictures, and little attention has been paid, so far, to the study of the generalization capability of these systems between different smartphone models.

The aim of this study is to describe, as a proof of concept, the use of an AI algorithm embedded in a mobile app able to read CrAg LFA and interpret whether the result is positive, negative, or invalid in an objective and automatic manner. Moreover, we also determine if the proposed system is able to quantify the signal test intensity of the CrAg LFA which in an ideal situation could be used for the characterization of the syndrome as well as for the follow-up of the diseases after the treatment initiation.

## 2. Materials and Methods

### 2.1. Experiment Design and Processing Pipeline

**Figure 1:**
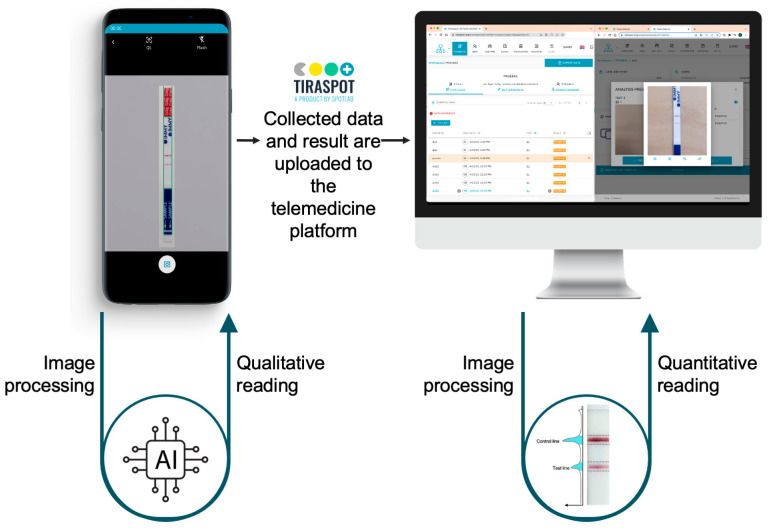
The proposed digital system is composed of: (1) a mobile application for POCT digitization, empowered with an integrated AI algorithm for result interpretation, and (2) a web platform for data collection with a quantitative readout algorithm.

The proposed AI-assisted system for POCT result interpretation and reporting is presented in Figure 1. The process is as follows: first, the CrAg LFA is digitized with the mobile phone by taking a picture using an app that guides the user to obtain standardized images with enough quality to be analyzed. The AI algorithm integrated into the mobile app suggests the result to the user, who can confirm or edit it. The POCT pictures, together with the corresponding relevant data, associated with the sample, are transferred to the web platform, where the user can review all the information, which may include CrAg concentration estimations given by additional quantitative AI models.

### 2.2. Sample Preparation and Data Acquisition

Fifty-two *Cryptococcus* antigen concentrations (ranging from 0.25 to 5000 ng/mL) prepared from the positive control provided by the kit as well as negative controls (no CrAg) were tested in duplicate during at least three different days with the CrAg LFA (IMMY, OK, USA). All dilutions were performed in standardized human sera (Merck, Sigma-Aldrich, Madrid, Spain). Some pictures for some concentrations were taken on a fourth experiment as well as some concentrations were photographed more than two times, for different reasons, which means, the pictures set was not uniform for the 53 antigen concentrations tested.

To assess the variability, each POCT was digitized and photographed, at least twice, by two different smartphone models with different technical capacities. One of the smartphone models is considered to be low-mid range (Motorola Moto E6), while the other is considered to be upper-midrange (Samsung Galaxy S9). Both devices are equipped with a ≥12 MPx camera.

In total, 361 CrAg LFA were digitized, forming a set composed of 1500 images. All photographs were obtained using an artificial light source of approximately 6000 K neutral temperature.

To generate the ground-truth which means to annotate the result of every LFA to build the training and validation sets of the AI algorithm, each POCT was visually interpreted by, at least, two different observers and classified as positive or negative. In those cases where there was a discrepancy between the two observers, a third one decided which was the final result after a careful reading of the questioned picture.

To evaluate the quantitative readout algorithm, only 318 LFAs were considered resulting in a database that ensures that all concentrations are equally represented, with a total of 1272 images (53 CrAg concentrations × 2 times × 3 different days × 2 pictures × 2 mobiles = 1272 images).

### 2.3. Mobile Application and Cloud Platform

All POCTs were digitized using the TiraSpot mobile app (Spotlab, Madrid, Spain). To take a correct picture of the POCT, TiraSpot displays a mask with the exact geometry and morphology of the CrAg LFA. Once the picture is taken, the app shows the image asking the user to confirm a correct acquisition (aligned with the mask and on focus). Configuration of the app can be freely adapted to the specific needs of the health system and can include any other relevant data required from a simple test determination to many variables to fill an observational database. The system can work without Internet access and once the device is connected all the information is uploaded to the cloud database.

### 2.4. Artificial Intelligence Algorithm for POCT Qualitative Reading

**Figure 2:**
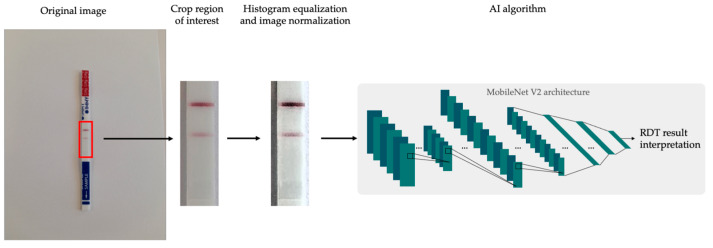
Image processing pipeline. First, the region of interest is cropped, which is then preprocessed and introduced into the CNN algorithm to predict the POCT result.

The AI algorithm was designed to interpret the test as positive, negative, or invalid. Once the valid picture of a CrAg LFA strip was taken and approved by the user, the algorithm pre-processes the original photograph by cropping the image to extract only the area where the control and test bands are, discarding the rest of the zone. This cropping is carried out based on the position that the LFA strip has in the mask presented to the user. A successful cropping process is dependent on a correct alignment between POCT and masks at the time of taking the photograph. Then, image normalization and contrast enhancement techniques are applied to the cropped image to highlight the faint bands and increase sensitivity. Finally, the processed image is introduced into a convolutional network (MobileNet V2 [19]) to predict the result. The entire image processing pipeline is shown in Figure 2. This network architecture has been specifically designed to be lightweight so it can be integrated into smartphones in a computationally efficient manner.

### 2.5. Artificial Intelligence Model for POCT Quantitative Reading

As we tested fifty-three *Cryptococcus* antigen concentrations (ranging from 0.25 to 5000 ng/mL) we decided to see if there was any correlation among the different concentrations and the signal intensity of the positive test in the LFA strip. An image analysis pipeline was developed to identify control and test lines of CrAg LFA and quantify their intensities. Since small variations in illumination intensity (low, medium, high) and color temperature (cold, warm) can affect the intensity and appearance of POCT lines, we applied brightness and color correction techniques to the images. This correction makes the quantification process robust against variations introduced when using different lighting scenarios or different mobile models.

**Figure 3:**
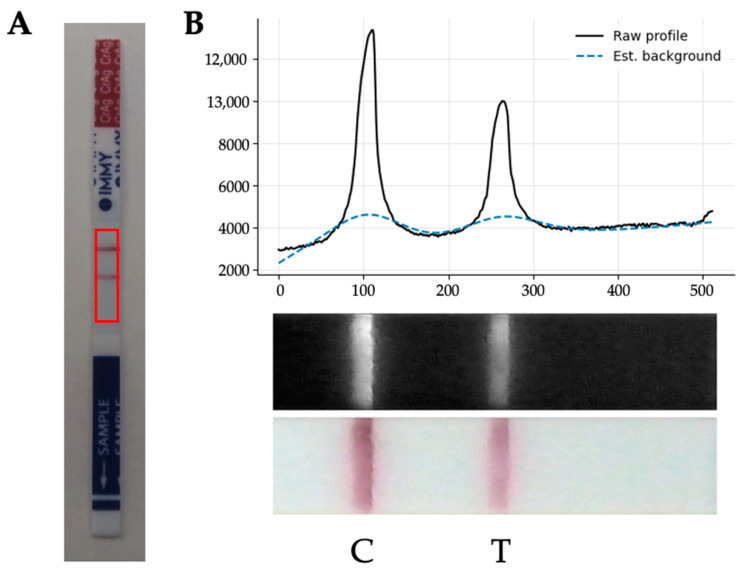
(**A**): Sample positive CrAg LFA, with the area of interest highlighted in red. (**B**): Control (C) and test (T) line quantification, showing the raw profile (black line) and estimated background (blue dashed line) of a representative positive POCT.

Once the image is pre-processed, the region of interest of the POCT result window is automatically detected and cropped using computer vision methods. With this purpose, we first detect both the upper red and lower blue areas of the CrAg LFA. Once these color areas are segmented, we identify the region of the result window, which is finally cropped. This cropped image is converted to grayscale and a one-dimensional signal profile is calculated as the average intensity along the short axis of the image. Then, the profile background is estimated (by using the asymmetric least squares smoothing method) and corrected, for normalizing among different ambient lighting conditions (see Figure 3). Control and test line intensities are then quantified from the corrected signal profile, and normalized test intensity (ratio between test and control line) is computed.

Regression modeling was used to explore the relationship between normalized test band signal intensity obtained by the quantification algorithm and CrAg concentration.

### 2.6. Validation Protocol and Statistical Analysis

Regarding the qualitative readout algorithm, the image dataset was randomly split so 2/3 of the available images were used for training the algorithm, while the remaining 1/3 of the images were used for validation purposes. To generate an independent validation set we ensure that pictures taken from the same LFA were not used for both training and validation. The performance of the AI algorithm for qualitative LFA result interpretation was assessed in terms of sensitivity (SN), specificity (SP), the area under the ROC curve (AUC), and the overall accuracy (ACC) in the validation dataset, by comparing the visual interpretation (consensus among different observers) and the AI predictions. The mean, standard deviation, and 95% confidence intervals (CIs) were properly calculated. The DeLong test [20] was used to compare the AUC and evaluate whether there is a difference in the performance of the AI algorithm for different smartphone models.

On the other hand, the regression model that relates the signal intensity of test lines and antigen concentration was evaluated by calculating the Pearson correlation coefficient (r).

## 3. Results

### 3.1. Real-Time AI-Assisted Reading of Qualitative CrAg LFA

Of the 1500 images used for training and validating the algorithm, a total of 29 images needed to be reviewed by a third observer due to a disagreement between the two initial observers. All of these images corresponded to CrAg LFAs inoculated with low CrAg concentrations (≤6 ng/mL). In all but one of these images, the third observer decided that they were positive although the test band was very faint being hardly visible. Of the 29 images that required the intervention of a third observer, 20 of them belonged to the training dataset, while the remaining 9 belonged to the validation dataset.

The independent validation set (1/3 of all available images) was composed of 502 images obtained after reading 120 different POCTs from the total 361 POCTs included in the study. As stated in the material and methods section, each POCT was photographed by two different smartphone models (Samsung S9, Motorola Moto E6). The POCTs used for evaluation were different from the ones used for training the algorithm.

**Figure 4:**
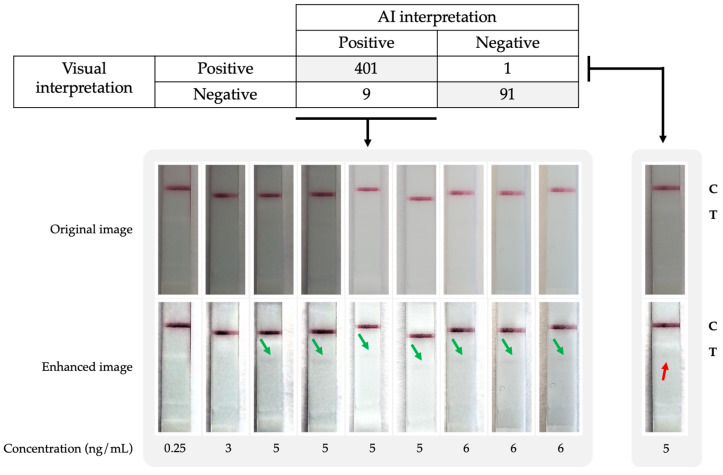
Confusion matrix between visual and AI interpretation. Images representing the discrepancies between analyses are also shown: green arrows indicate correctly identified weak bands by the AI not identified by the visual analysis. The red arrow represents a false negative case where the AI algorithm did not detect a visible weak band.

The performance of the algorithm (SN, SP, AUC, ACC) for the automatic reading of the CrAg LFA is detailed in Table 1, which also shows the performance for each of the smartphone models independently. We compared the AUC (DeLong test) for both smartphone models and no statistically significant difference was found (*p*-value = 0.93). The confusion matrix, as well as the POCT photographs of discrepancies between visual interpretation and AI predictions, are shown in Figure 4. Nine images were classified as positive by the AI algorithm while the users interpreted them as negative. A new inspection of the discrepancies revealed that seven of the nine images rendered as negative by visual inspection were, in fact, positive (Figure 4: green arrows). Then, the lower limit of AI detection was established between 5 to 6 ng/mL which is lower than the visual one. Only one false negative case was observed (at 5 ng/mL), where a very weak band was visually identified while the AI was negative (Figure 4: red arrow).

Additionally, it was observed that out of the nine evaluation images where there was a discrepancy between the two observers, the AI correctly predicted the outcome and agreed with the third observer in eight out of nine cases. This fact proves that the algorithm is indeed able to provide an answer that could reduce the variability and discrepancy between observers.

The AI algorithm was integrated into the acquisition mobile app enabling a real-time execution of the model (AI model runs on the mobile itself and no Internet connection is required). The user obtained the result in 121 ms (95% CI [120–123]), demonstrating that the AI App feeds back to the user at the time of image acquisition. The Supplementary Video S1 shows a video of a real digitalization operation and real-time interpretation by the AI, helping the user to interpret the CrAg LFA result.

### 3.2. Quantitative Signal Measurement of CrAg LFA

We included 318 digitized CrAg LFAs for the quantitative assessment to ensure the 53 concentrations were equally represented, which means the analysis of two replicates for each concentration during three different days. All replicates were photographed twice by two different smartphones counting a total of 1272 images (24 images per concentration).

**Figure 5:**
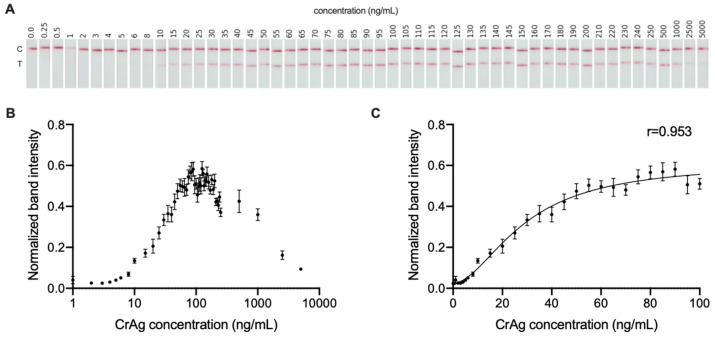
(**A**) Representative CrAg LFA images with different antigen concentrations. (**B**) Dose-response curve for the CrAg LFA, showing hook effect after 150 ng/mL approximately, and dynamic range being from 0 to 100 ng/mL approximately. (**C**) Quantitative results showing the fitting curve using regression analysis which relates CrAg concentration with signal intensity.

Signal intensity was measured and quantified on the whole dataset composed of 1272 images. Figure 5A shows representative examples of POCTs inoculated with each of the 53 concentrations tested. Figure 5B shows the dose-response curve for the CrAg at the dynamic working range of the CrAg LFA which was between 0 and 100 ng/mL. The limited range of the dose-response curve was due to a postzone effect (or high-dose hook effect) starting with concentrations greater than 150 ng/mL, which lead to a contradictory decrease in the positive test line intensity as the concentration of the antigen increases. Four parameter logistic (4PL) regression was found to be the model that best related signal intensity and CrAg concentration. Figure 5C shows the fitted 4PL regression model, which obtained a Pearson correlation coefficient of 0.953 (95% CI [0.946–0.959]) between the band signal intensity versus CrAg concentration within the dynamic range.

On the other hand, we evaluated the capability and assessed the robustness and generalization capability of our quantification method between two different smartphone models. To test this, we used only the images obtained by one smartphone model to fit the regression model, and then, the obtained fitting curve was evaluated with the pictures taken with the other model. We did the same process with the other phone model. Moreover, we also performed the experiment using only the images of the same model for both fitting and evaluation. Table 2 shows the results of this evaluation through the comparison of Pearson coefficients (and its corresponding 95% CI), and no significant differences were found between any of the possible pairs of experiments (all *p*-values > 0.1) which means the generalization capability of the system among different devices.

## 4. Discussion and Conclusions

Fungal disease has been neglected for many years. WHO has recently established a priority list of fungal pathogens that will hopefully help to reduce the two million deaths they cause each year, mainly in low- and middle-income countries [21]. Access to the diagnosis of fungal diseases is essential to provide appropriate treatment to patients, but there are many barriers to rapid access due to the lack of healthcare workers trained in these diseases. Therefore, there is an increasing need to develop systems that are effective, fast, inexpensive, accessible, and capable of being used by healthcare workers without specialized training.

POCTs are being increasingly incorporated into the diagnostic portfolio for infectious diseases [22]. They are extremely useful to be set up with limited resources because of their low price, lack of sophisticated equipment for their operation, as well as the rapid response obtained, which is critical for life-threatening diseases [23]. However, the lack of healthcare workers trained in their use and interpretation of results limits the wide use of the POCT tests. Moreover, the test results are usually written down on paper which makes it cumbersome to report the results to the competent health authorities in an efficient way as well as retrospectively access and check results if needed. All of these issues are barriers to estimating the real burden of fungal disease, as well as to calculating the needs of antifungals needed for the treatment. This is especially important if they are not registered in the country which ultimately hampers the development of public health programs to allocate the appropriate resources to control the specific disease problem.

The use of an AI-app-based specifically designed to read and interpret POCTs implies an automatic, objective, and standardized procedure decreasing subjective interpretation as well as, covering the inherent limitations associated with the visual reading of the test—different workers, with different training and with different visual acuity. These systems provide an objective judgment, as well as help to standardize in the real world the innate sensitivity and specificity of the POCT approved by the regulatory organizations based on data obtained through experiments performed in highly controlled situations. Previous work has proposed the combined use of a mobile app and AI algorithms to digitize and interpret POCT results that have been shown to be usable with a variety of different SARS-CoV-2 POCTs [24]. In addition, current interpretation of POCTs is usually only qualitative, and in some specific situations, the quantification of the analyte tested can be of further help to a better assessment of the disease risk or even distinguish among different stages of it, which can be critical for the management of the patient.

On the other hand, smartphones are now a global tool that, among other uses, have shown great capability of data collection and transmission. AI algorithms embedded in mobile phones can be transformative for the diagnosis of diseases especially when high sensitivity and specificity POCTs are available, as is the case of CrAg LFA test. For life-threatening diseases, it is key for the patient to be diagnosed and treated as soon as possible. The use of this approach decreases the need for training healthcare workers offering them both reading systems, visual and automated. Moreover, the digitization of the LFA test allows quick communication with the physicians in charge of the patient, rapid instauration of treatment increasing chances of survival, second opinions as well as storing the results and the patient’s data in a database in the cloud which facilitates, among other things, the estimation of the burden of the disease investigated.

In this work, we have described the design, development, and validation of an AI-based mobile application able to automatically read CrAg LFA. The proposed system, which does not require additional hardware and can be installed from regular app marketplaces in any mobile phone, not only is capable of performing a qualitative reading of the POCT (positive, negative, or invalid) but is able to predict antigen concentration based on the signal intensity of the band on the POCT, providing a quantitative reading. All AI results along with POCT images are sent to a cloud platform that allows for case identification, quality control, and real-time monitoring.

Our qualitative reading algorithm shows an area under the ROC curve (AUC) of 0.997 (95% CI: [0.992–1]) when comparing the visual interpretation of CrAg LFAs and AI predictions. Moreover, the algorithm improves the limit of detection compared to the visual interpretation, as it detects weak bands (associated with low concentrations, ranging from 5 to 6 ng/mL) that are not usually detected by the eye. Having a lower limit of detection will allow us to identify patients that need to be fully diagnosed and treated according to the results obtained which will decrease the mortality rate of this opportunistic infection. For life-threatening diseases such as cryptococcal meningitis, false negative results are of serious consequence. False positive results can be managed accordingly with other parameters related to the specific disease.

On the other hand, we have demonstrated the ability to predict CrAg concentration by just relying on a picture of the POCT by measuring signal intensity. We found a strong correlation between signal intensity and antigen concentration, with a Pearson coefficient (r) of 0.953 (95% CI: [0.946–0.959]) but for a limited working range established between 0 and 100 ng/mL because of the postzone effect. The postzone effect, already described with this POCT [25,26], impedes the real quantification of antigen especially when the samples have very high concentrations which, usually, are related to meningitis. However, strategies can be devised such as performing an additional dilution to determine whether a particular sample is being affected by the hook effect or not.

In conclusion, we have proven that a qualitative test can be turned into a quantitative metric with a proper AI algorithm and that performance between different smartphone models was similar.

Future work includes further assessments of this approach using real biological samples from patients to provide a clinical evaluation, and the use of tests without postzone effect to clinically evaluate the possibility of using qualitative tests together with AI quantification algorithms to predict meningitis, adjust antifungal treatment, and consequently, improve patient survival.

This approach has several advantages as it provides an easy-to-use AI-based mobile app for automatic and objective result interpretation of the CrAg LFA, which is able to reduce reading variability among different health care workers, providing an important help in environments where training is limited and the replacement of health care workers is frequent. On the other hand, the mobile app is connected to a web platform, where all results can be safely stored allowing burden estimates or large epidemiological studies eliminating the need for retrospective analysis of lab records, and providing a valid tool for quality control processes.

## Figures and Tables

**Table 1:** Performance of the AI algorithm for qualitative POCT interpretation for all validation images. The performance of each smartphone model is also reported.

	SN [95% CI]	SP [95% CI]	AUC [95% CI]	ACC [95% CI]
All	0.998 [0.993–1]	0.910 [0.885–0.935]	0.997 [0.992–1]	0.976 [0.963–0.989]
Samsung S9	1 [1–1]	0.920 [0.887–0.953]	0.997 [0.990–1]	0.976 [0.957–0.995]
Motorola Moto E6	0.995 [0.986–1]	0.900 [0.863–0.937]	0.997 [0.990–1]	0.976 [0.957–0.995]

**Table 2:** Evaluation of different fitting curves (Pearson coefficient, r) when trained and tested on images coming from different smartphone models.

		Models Used for Evaluation		
		Motorola E6	Samsung S9	Both
Model used for fitting	Motorola E6	0.961 [0.952–0.968]	0.957 [0.947–0.965]	0.953 [0.946–0.959]
	Samsung S9	0.96 [0.951–0.967]	0.957 [0.947–0.965]	0.953 [0.946–0.959]
	Both	0.96 [0.951–0.967]	0.957 [0.947–0.965]	0.953 [0.946–0.959]

## Data Availability

The data supporting the results of this study will be made available upon reasonable request from the corresponding author.

## Supplementary Materials

The following supporting information can be downloaded at: https://www.mdpi.com/article/10.3390/jof9020217/s1. Video S1: Real-time AI and mobile app video demo.
